# Branchial cleft cyst and branchial cleft cyst carcinoma, or cystic lymph node and cystic nodal metastasis?

**DOI:** 10.1017/S0022215122001293

**Published:** 2023-01

**Authors:** P Stefanicka, M Profant

**Affiliations:** Department of Otorhinolaryngology – Head and Neck Surgery, Medical Faculty, Comenius University in Bratislava, Slovakia

**Keywords:** Branchioma, Cysts, Head And Neck Neoplasm, Lymphatic Metastasis, Carcinoma, Squamous Cell

## Abstract

**Background:**

Lateral cervical cysts are usually considered as of branchial cleft origin, despite many studies showing that branchial cysts do not arise from the remnants of the branchial apparatus. In the same way, some authors still consider that a true clinicopathological entity such as ‘branchial cleft cyst carcinoma’ could exist, at least in theory. Despite insufficient evidence in support of the branchial theory, a number of publications continue to emphasise this concept.

**Methods:**

A literature review of articles in Medline and PubMed databases was carried out to retrieve papers relevant to the topic.

**Results and conclusion:**

The evidence from lateral cervical cyst studies and knowledge about cystic metastasis of Waldeyer's ring could be applicable for both diagnoses. Terms such as ‘branchial cleft cyst’ and ‘branchial cleft cyst carcinoma’ are confusing and misleading, and it is questionable as to whether their usage is still tenable.

## Introduction

The majority of cystic lesions in the lateral aspect of the neck are believed to arise from remnants of the branchial arches, and for this reason they are referred to as branchial cysts. This ‘branchial theory’ was suggested in 1832 by Ascherson (as cited in Golledge and Ellis^1^), who associated cervical fistulae with the arches and clefts, and which led to him having a similar assumption regarding cervical cysts, without any real evidence. Several theories have been proposed regarding the cause of branchial cleft cysts; however, as embryological investigations have proceeded, the concept of their origin from branchial clefts, the precervical sinus and the thymopharyngeal duct has been rejected.^2–4^

Later histological studies showed a close relationship between the cysts and lymph nodes, and cystic transformation of cervical lymph nodes was proposed as an aetiology of ‘branchial cysts’. Because of the characteristic histological appearance of the ‘branchial cyst’, the purely descriptive term ‘lateral lymphoepithelial cyst’ was recommended.^2,3^ The stimulation to a cystic transformation of the lymph node was explained by trapped epithelium, in line with ‘inclusion theory’.^2^

Further studies pointed out the remarkable similarities between ‘branchial cysts’ and tonsillar crypts in terms of histological appearance, ultrastructural make up,^4–6^ immunohistochemical analysis^7^ and cytokeratin expression profile.^8^ These similarities, and this apparent relationship between lateral cervical cysts and tonsillar crypts, supported the tonsillar crypt epithelium as a source of inclusion. According to this, epithelium from the tonsillar crypt could enter the cervical lymph nodes and cause cystic alteration, resulting in a lateral cervical cyst.^1,8^

If the ‘branchial cyst’ is a cystic lymph node, is the ‘branchial cyst carcinoma’ at least a hypothetical entity? Recently, it has become clear that cystic metastases, which have often been mistaken for branchial cleft cyst carcinomas, are from primary carcinomas in the tonsillar tissue of Waldeyer's ring.^9–11^

Many reports regarding primary branchial cleft cyst carcinoma have failed to provide sufficient evidence to distinguish this entity from nodal metastases arising from unrecognised primary tumours.^12–14^

Reports identifying the transition from normal epithelium to malignancy, through dysplasia and in situ carcinoma, appeared for some to favour a branchial cleft carcinoma diagnosis. However, in many publications, these findings were linked with the detection of the primary tumours, and subsequently a diagnosis of branchiogenic carcinoma became unacceptable.^10,11,15,16^

Human papillomavirus (HPV)-associated oropharyngeal carcinoma has been identified as a distinct entity, with clinical, histological, molecular and prognostic characteristics distinct from smoking-associated squamous cell carcinomas (SCCs) of the head and neck.^17–19^ A series of studies have shown the involvement of HPV infection in the formation of cystic node metastasis.^17,20,21^

This review aimed to provide an overview of the present data regarding these two diagnoses.

## Branchial cleft cyst or cystic lymph node?

Lateral cervical cysts were first described by Hunczovsky in 1785 (as cited in Golledge and Ellis^1^). Since that time, several aetiological theories of these cysts presenting in the lateral aspect of the neck have been outlined. In 1832, Ascherson introduced the branchial theory of cyst development.^1^ He suggested that cervical cysts were the result of the imperfect obliteration of a pharyngeal cleft. However, this theory was based only on Ascherson's impression that lateral cervical cysts developed at the site occupied by the branchial apparatus in the embryo. In 1886, His (as cited in Golledge and Ellis^1^) considered that branchial fistulae were related to the cervical sinus, rather than the pharyngeal clefts or pouches, and similarly equated the development of branchial fistulae with lateral cervical cysts. Wenglowski (1912, as cited in Golledge and Ellis^1^) showed that pharyngeal cleft tissue was not represented in any adult tissue inferior to the hyoid bone. Thus, no cyst lying below this level could be derived from the branchial cleft, and he proposed the theory that the lateral cervical cyst is the result of incomplete obliteration of the thymopharyngeal duct.^1–3^

King^3^ reviewed current hypotheses and studied the clinical and histological features of 76 ‘branchial cysts’. He concluded that the cysts had no direct relationship with any of the structures in the early embryo. Because embryological investigations indicated their inadequacy and because the cysts did not show any constant features that might support these speculations, King rejected the congenital hypothesis. He emphasised the relationship of the cysts to the lymph nodes and lymphoid tissue, and suggested this as an alternative hypothesis. In light of the characteristic histological appearance, he preferred the purely descriptive term ‘lateral lymphoepithelial cyst’. However, King supposed that the epithelial lining of the cysts originated from the endothelium of the nodes, and he did not describe the mechanism of formation of these cysts.^3^

The close relationship between these cysts and lymphoid tissue was supported by Bernier and Bhaskar's study.^2^ They observed that, in 452 of 468 cases, the cysts were surrounded by lymphoid tissue and lined by stratified squamous epithelium. If the cyst was large, the lymphoid tissue was compressed to a narrow peri-epithelial zone. In other cases, in which the cystic lesion had involved only part of the node, the nodal character of the lymphoid tissue was easily recognisable. Bernier and Bhaskar's investigation showed that possibly 96 per cent of branchial cysts are cystic lymph nodes. The majority of branchial cysts stem from cystic alteration of epithelium trapped in cervical nodes. They supported the assumption that the epithelium within the cervical lymph nodes is glandular in origin. In concordance with King,^3^ they suggested the term ‘benign cystic lymph node’ or ‘benign lymphoepithelial cyst’.^2^

Later, Maran and Buchanan^4^ reviewed branchial cysts, sinuses and fistulae, with special reference to their origin, clinical features, pathology and treatment. Material was collated from over 700 cases reviewed in the literature, a personal series of 42 cases and a retrospective series of 90 cases. The detailed microscopic appearance of the ‘branchial cyst’ showed squamous epithelium, with surrounding lymphoid tissue closely resembling tonsillar tissue. The cystic alteration occurs in epithelium that is believed to be entrapped in nodes of the neck during embryogenesis. However, the evidence was not proven, and these authors pointed out that similar lesions are not seen in lymph nodes received as surgical specimens from other anatomical sites.^4^

Other authors demonstrated that lateral cervical cysts had a complex structure composed of stratified squamous epithelium, with an intimate association with tissue resembling a lymph node, whereby the node is part of the cyst wall. The branchial sinuses and fistulae had a totally different structure.^22^

Previous investigations had already demonstrated that branchial cysts have many similarities to palatine tonsils when their linings are studied by light microscopy, scanning and transmission electron microscopy, and enzyme histochemistry. These results show that the epithelium of the branchial cysts is a specialised tissue. It is not simply an inactive lining separating the cyst lumen from lymphoid tissue. The epithelium is specialised in that it takes the form of the crypt epithelium of the tonsils, which is believed to be involved in the uptake of antigens from the gut and the presentation of antigen to cells of the lymphoid system.^5,6^

Crocker and Jenkins^7^ studied the organisation of the branchial cysts in great detail, applying immunohistochemical procedures to a series of 25 specimens, with reference to both the lymphoid elements and the lining epithelium. This study showed certain similarities between the epithelial elements of branchial cysts and palatine tonsils. The orientation of lymphoid follicles in branchial cysts (their mantle zones are directed towards the epithelium) is analogous with their direction towards the crypt epithelium of tonsils and the marginal sinuses in lymph nodes. The authors might have therefore concluded that the lymphoid tissue in branchial cysts is arranged normally in a functional sense. Certainly, the presence of the sinuses themselves provides strong evidence in favour of the origin of branchial cysts from lymph nodes.^7^

Golledge and Ellis^1^ studied four aetiological theories of lateral cervical cysts, and the evidence for and against these theories was discussed. They found that early descriptions of branchial cysts were based on the anatomical site and the consistency of the cyst, rather than being a histological diagnosis. Of the four hypotheses regarding the aetiology of the lateral cervical cysts, the evidence strongly favours the cystic transformation of the cervical lymph node. The principal support for this aetiology comes from histological studies.^1,4,7^ Golledge and Ellis^1^ also scrutinised the medical records and histology of 20 patients who had undergone excision of lateral cervical cysts. Of particular interest was the finding that, in all cases, the cyst wall contained lymphoid tissue with recognisable lymphoid follicles. In no case was a tract or cord connecting the cyst to the skin or pharynx noted.^1^

Wild *et al*.^23^ showed, according to the particular pattern of keratin polypeptides, that the inner lining of cervical cysts was homologous to upper digestive tract squamous epithelia, specifically oropharyngeal crypt epithelial cells. Later, Wild and colleagues analysed the epithelial lining of lateral cervical cysts for keratin polypeptide composition.^8^ The keratin phenotype expressed in branchial mass epithelia was found to be homologous to the profiles obtained for the squamous epithelium of corresponding palatine tonsils. The presence of particular keratin members strongly indicates that branchial mass inner lining derives from keratinocytes that are programmed to form a stratified squamous epithelium. On the basis of the anatomical, clinical and biochemical findings, Wild and colleagues proposed that a lateral cervical cyst could be an acquired condition, most likely resulting from Waldeyer's ring crypt epithelial cells, which may settle and transform the lymph node to form epithelium capable of mimicking the crypts of the palatine tonsils.^8^

Golledge and Ellis^1^ supported the palatine tonsil as a source for the included epithelium. They agreed that epithelium from the palatine tonsil enters the cervical lymph nodes during adult or infant life, and causes cystic transformation resulting in a lateral cervical cyst.

In one clinical study, the authors carried out an experiment to evaluate lymphatic drainage from the oropharynx to the lateral cervical cysts. By injecting the blue stain into the tonsillar region of patients with a lateral cervical cyst, the lymphatic channels of the oropharynx were identified. The distribution of the stain within the capsule of the cyst was observed during the operation. This experiment indicates that lateral cervical cysts may be interpreted as being cystic lesions of a lymph node.^24^

Cao *et al*.^25^ described the distribution of p16 staining in 37 benign lymphoepithelial cysts. The most intense p16 staining in lymphoepithelial cysts was seen in a reticulated epithelium, distributed evenly as in the reticulated crypts epithelium in tonsillar tissue. Staining of the tonsillar crypt epithelium was focal and generally limited to clusters of non-keratinising cells. P16 staining was not observed in the stratified squamous epithelium that covered the surface of the tonsils. This immunohistochemical finding shows an apparent relationship between the cervical lymphoepithelial cyst and the tonsillar crypt. In some instances, P16 expression appears to reflect the intrinsic property of a specialised squamous epithelium.^25^

Transmission electron microscopy of the normal tonsil shows many interesting features of the specialised reticulated epithelium in the crypt spaces. The tonsil crypt is the specialised area of interface between the epithelium, lymphocytes and vessels. The basement membrane is absent in many places, and numerous capillaries are adjacent to the epithelial cells, regardless of whether or not the cells have a basement membrane. Human papillomavirus integration was shown to occur in these epithelial cells. Malignant epithelial cells could therefore gain access to vessels without an intervening basement membrane. Moreover, surrounding each lymphoid follicle, efferent vessels pass to the deep cervical nodes. All of these could help to explain the early and common occurrence of cervical nodal metastases from tonsillar crypt carcinomas.^26^

Hypothetically, these cryptal epithelial cells could easily escape to the lymph node similarly without malignant transformation.

## Branchial cleft cyst carcinoma or cystic nodal metastasis?

Branchial cleft cyst carcinoma is defined as a cancer arising from cells within the cyst wall of an existing branchial cleft cyst.^27^ In 1882, Von Volkmann^28^ was the first to suggest that some cervical cancerous tumours might arise in the vestigia of branchial clefts. Von Volkmann's belief that the cervical tumours in his three cases arose primarily in branchiogenic remnants was based solely on the fact that he was unable to discover a primary lesion after direct visual and digital examination of the oral cavity and pharynx. This theoretical presumption became widely accepted during the first half of the twentieth century, and many publications demonstrated branchial cleft cyst carcinomas, the diagnosis of which was based on similar principles with insufficient evidence. In 1893, Sutton commented that, in most cases, these gland masses are secondary to epithelioma originating in recesses of the pharynx or nasopharynx, and the theory that they arise in remnants of branchial clefts is pure fiction (as cited in Martin *et al*.^29^). Willis, in 1934, supported this belief, and stated that branchiogenic cancer should be tolerated neither as a clinical diagnosis nor as a histological finding on surgical material.^29^

In 1950, Martin *et al*.^29^ published a landmark paper on branchiogenic carcinoma. They realised that the diagnosis of branchiogenic cancer is too often and too loosely made, and accordingly established four criteria to fulfil the requirements for even a tentative branchiogenic cancer diagnosis. They designated the fourth of these criteria, a histological demonstration of a cancer developing in the wall of an epithelial-lined cyst, as the most important in the confirmation of branchiogenic cancer. After reviewing the 250 cases published up to that time, they were able to eliminate all but 3 instances. They then added 15 cases of their own. Neither the cases reviewed nor the 15 cases added by Martin *et al*.^29^ satisfied the fourth criterion. They did not find histological criteria which could possibly differentiate metastatic cancer from that arising in branchiogenic vestigia. Martin *et al*.^29^ concluded that the actual existence of a clinical entity deserving the specific term ‘branchiogenic cancer’ is entirely hypothetical. A definitive diagnosis of branchiogenic cancer cannot be made on a histological basis.

Khafif *et al*.,^12^ in 1989, reviewed the English-language literature concerning branchial cleft cyst carcinoma since the report of Martin *et al*.,^29^ and found 67 cases. Only eight cases satisfied Martin and colleagues’ third criterion of five-year follow up without evidence of a primary carcinoma elsewhere. The authors argued that this third criterion cannot often be satisfied, as patients may die of unrelated causes before the five-year period elapsed, and many patients receive post-operative irradiation that may control an occult primary malignancy. In place of Martin and colleagues’ third criterion, they suggested two additional criteria: (1) identification of transition from a normal squamous epithelium of the cyst to a carcinoma; and (2) absence of any identifiable malignant tumour after exhaustive evaluation of the patient.^12^

Other authors supported Khafif and colleagues’ modified criteria for diagnosing branchiogenic cancer as clinically more practical, because a definitive diagnosis can be made immediately, based on the histopathological findings of a progression from normal cyst lining epithelium through dysplastic epithelium to invasive carcinoma. New cases of branchial cleft cyst carcinoma with this histological appearance were reported, but the evaluation of a potential clinically occult primary tumour was not exhaustive (missing appropriate biopsies or tonsillectomies).^13,14,30^

Micheau *et al*.^10^ have already concluded that so-called branchiogenic carcinomas are actually cystic metastases in the neck from tonsillar primary. They presented reports on 21 such cases, and showed that cystic metastases from tonsillar SCCs have often been mistaken for either primary SCCs of branchiogenic origin or branchial cleft cysts. The distinctive histological features of cystic metastases reviewed after correct identification can lead to the discovery of an unsuspected primary lesion. Moreover, they found that all of Martin and colleagues’ criteria can be applied to cystic metastasis in the neck if the primary lesion was not identified for at least five years. The majority of primary carcinomas were discovered within three years after initial treatment of the neck.^10^

Mallet *et al*.^11^ presented two cases with cystic masses with the histological appearance of a transition from normal squamous epithelium to dysplasia, and carcinoma in situ of the cyst to carcinoma. In both cases, the primary carcinoma was identified on tonsillectomy and endoscopy, with a biopsy of the tongue base. Although the histological finding satisfied the histological criterion for the diagnosis of a branchial cleft carcinoma, rapid detection of the primary lesion made no other diagnosis possible. The authors concluded that Martin and colleagues’ criteria are, in practice, poorly adapted to the diagnosis of a branchial cleft carcinoma over a cystic metastasis of an SCC, and should be abandoned. Consequently, it is not likely that a branchial cleft carcinoma will ever be diagnosed in such conditions. However, the demonstration of a complete cystic tract does indeed plead for a branchial cleft carcinoma.^11^

Thompson and Heffner^9^ reviewed 136 cases of cervical cystic SCC, and none of the cases involved a branchiogenic carcinoma. They defined the histological features of ‘typical’ cystic cervical SCC metastasis, in which the overall histological appearance in many areas was very bland, recapitulating the normal squamous to transitional-type epithelium identified in tonsillar crypts or occasionally identified in branchial cleft cysts. These ‘typical’ cystic metastases, almost always mistaken for carcinoma arising in a branchial cleft cyst, are almost always from primary carcinomas in the tonsillar tissue of Waldeyer's ring. Because tonsillar carcinoma cystic metastases can have areas lining the cyst that are practically benign in appearance, this can mimic SCC arising in the branchial cleft cyst with an already mentioned transition from normal squamous epithelium to carcinoma. Thompson and Heffner reported cases in which the primary tumour was not discovered for periods longer than 5 years (up to 11 years), but then was discovered. This indicates that this type of primary tumour can be indolent in its growth, which could be explained by the nature of the tonsillar crypt lymphoepithelium from which they arise. The crypt epithelium is normally so intimately associated with lymphoid cells that the metastatic epithelium is perfectly suited to the microenvironment furnished by the lymph node. This interaction may account for the well-developed cystic formations, as the carcinoma imitates the parent crypt epithelium that normally invaginates into lymphocytic tissue. Finally, these authors stated that an SCC arising from a branchial cleft cyst is a hypothetical entity that, from a practical clinical standpoint, does not exist.^9^

Currently, there is satisfactory evidence that branchiogenic carcinoma is in fact cystic metastasis from an oropharyngeal carcinoma, most commonly originating in the tonsillar tissue of the Waldeyer's ring, and not a true carcinoma arising in a branchial cleft cyst.^9–11,15,31^

Human papillomavirus targets preferentially the highly specialised reticulated epithelium that lines the tonsillar crypts.^18^

It has been shown that HPV-positive node metastasis is specific to oropharyngeal carcinoma, that there is an association of oropharyngeal carcinoma with a histologically identified cystic node metastasis, and that the radiographically identifiable cystic node metastasis is more likely to be HPV-positive. Together, this suggests the role of HPV infection in the formation of a cystic node metastasis.^21,32,33^

## Cystic nodal metastasis or benign cystic lymph node?

Isolated cervical cystic nodal metastasis can mimic a lateral cervical cyst, but differential diagnosis of this cystic cervical mass is almost impossible based on clinical or even radiological findings. In such cases, the primary tumour is clinically and radiographically occult. Hence, a cystic metastasis can be confused with a benign lateral cervical cyst, resulting in delayed diagnosis or the misdiagnosis of a cystic neck mass that turns out to be malignant.^34–37^

In a study attempting to determine radiographic criteria to differentiate between a benign and malignant cystic neck mass, the authors showed that a malignant cystic adenopathy tends to be more heterogeneous, and more likely to have septations, a poorly defined border and a cystic wall with surrounding fat stranding or inflammation.^36^ However, in that study, there was also a higher degree of overlap between the features seen in both benign and malignant cystic lesions: 31 per cent of patients with metastatic cystic nodes had benign-appearing cystic adenopathy, and, conversely, 38 per cent of patients with benign cysts had aggressive features mimicking metastasis.^36^ Moreover, another study failed to find an association between malignancy and radiographic variables such as heterogeneity, septations and stranding surrounding the cyst.^37^

Positron emission tomography/computed tomography (PET/CT) does not seem to be a reliable modality for identifying malignancy in adults with suspicious cystic neck masses. Ferris *et al*.^35^ showed that PET-CT does not add substantially to the diagnostic evaluation of potential malignancy in suspicious cystic neck masses in adult patients. However, Abadi *et al*.,^37^ in a series of 58 patients with single cystic neck lesions, found that fluorodeoxyglucose PET/CT has a high sensitivity (95 per cent) and negative predictive value (96 per cent); hence, malignancy could reliably be ruled out, albeit with a high frequency of false positive scans requiring further diagnostic investigation.

Imaging methods such as ultrasonography, CT, magnetic resonance imaging or PET can identify suspect features of malignancy, although their findings are often inconclusive, especially regarding radiographic occult primary tumours.^34–37^

Although fine needle aspiration cytology (FNAC) is recognised as the best initial test to diagnose a cervical mass, its usefulness in cystic neck lesions is much less than in solid masses. The false negative rate of FNAC in the diagnosis of SCC in cystic metastases exceeds 50 per cent.^38–40^ The main problem is the shortage of diagnostic cellular material. Therefore, FNAC may need to be repeated, optimally with ultrasonography guidance to direct the needle into any solid components or a cyst wall.^38^

Layfield *et al*.^39^ evaluated 19 cytological features to determine which were useful in the distinction of benign cervical cyst and cystic SCC metastasis. They found that a high nuclear cytoplasmic ratio, irregular nuclear membranes and small cell clusters were the most helpful features in this differentiation. However, they had to conclude that the distinction of a benign cyst from a cystic nodal metastasis is cytologically difficult, and that diagnostic accuracy remains imperfect.

A number of ancillary techniques (e.g. image cytometry, HPV analysis) have been proposed to evaluate fine needle aspirates, but in many cases sufficient cellular material is not available.^41–43^ Although the presence of HPV in a squamous-lined cyst would provide compelling evidence of its malignant nature and oropharyngeal origin, the absence of HPV may not be decisive in confirming the benign nature of the cystic neck mass. Furthermore, there is a lack of consensus regarding HPV testing of fine needle aspirates.^41,42,44^

An ultrasound-guided core biopsy is considered a safe and highly effective technique for diagnosing neck masses, and has recently become more widespread. This modality offers the advantage of preserving tissue architecture with increased tissue yield, resulting in fewer cases diagnosed by open biopsies. In comparison, ultrasound-guided core biopsy has demonstrated increased diagnostic accuracy, and has comparable complication rates to FNAC. However, repeat ultrasound-guided core biopsy has a low yield, and patients with non-diagnostic biopsy results should be considered for excisional biopsy.^45–47^

Because of the challenging distinction between cystic nodal metastasis and a lateral cervical cyst, some authors have recommended the routine use of frozen section at the time of excision. Some have shown that FNAC is far less reliable in the diagnosis of branchial cleft cyst than frozen section, with sensitivities of 75 per cent and 100 per cent, respectively.^48^

If the primary site is clinically occult, and imaging and cytological findings are inconclusive, surgical excision with thorough histological examination can be used to confirm the diagnosis. However, waiting for a final histological diagnosis of a carcinoma leads to delays in the detection of an occult primary tumour and delays in the appropriate treatment. If completion surgery is planned, then previous surgery of the neck potentially increases complications because of violated neck tissue; typically, in these cases, more aggressive treatment is recommended, which increases patient morbidity.^34,40,48–50^

Currently, there is general agreement regarding the management of a solitary lateral cystic neck mass in adult patients (especially those aged over 40 years): until proven otherwise, these patients should be presumed to have cystic nodal metastasis. The optimal solution in such cases is to perform surgical interventions in one stage. If the FNAC reveals SCC, it is possible to plan elective treatment, panendoscopy with direct biopsy of Waldeyer's ring, tonsillectomy, and neck dissection in one stage. If the FNAC is non-diagnostic, excisional biopsy of the cystic mass with a frozen section biopsy is recommended. If the frozen section biopsy finding is positive for SCC, panendoscopy, direct biopsies of Waldeyer's ring, tonsillectomy and neck dissection should be performed in one stage. This avoids patient anxiety and distress associated with mismanagement. In these situations, the patient should receive appropriate pre-operative counselling.^20,34,40,48,51–53^ In patients with a highly clinically suspicious solitary cystic neck mass who are aged over 40 years, super-selective neck dissection might be a reasonable approach, because in experienced hands it is a low morbidity procedure and is more accepted than incomplete resection in cases of SCC of the neck.^54,55^

## Conclusion

There is no evidence to support the idea that lateral cervical cysts are of branchial origin. On the contrary, the evidence strongly favours the development of lateral cervical cysts after the cystic transformation of a lymph node. There are studies that support the concept of a tonsillar crypt epithelium trapped in a cervical lymph node as the stimulus of this cystic lymph node transformation.^1,7,8^ Similarly, as cystic metastases from primary SCC in Waldeyer's ring simulate the growth behaviour and growth pattern of the primary tumour parent cell, cystic formation in a lymph node could be considered an intrinsic property of the keratinocytes of tonsillar crypt epithelium.^9,33^ The specific ultrastructural anatomy of the tonsillar crypts, with its excellent access to vessels, without intervening the basement membrane, and with an abundant lymphatic supply, may explain the pattern of early nodal metastases associated with small indolent oropharyngeal primaries. This anatomy may facilitate migration of both cancerous and normal non-cancerous cryptal epithelial cells, but the precise mechanism by which this occurs is less well explained.^1,26^

‘Branchial cleft cyst’ is really a cystic lymph node; this could elucidate ideas regarding a hypothetical entity such as ‘branchial cleft cyst carcinoma’, as the existence of ‘branchial cleft cyst carcinoma’ without a ‘branchial cleft cyst’ is impossible.

The formation of an isolated benign cystic lymphoepithelial lesion and cystic metastasis in the lymphatic drainage of the Waldeyer's ring appears unique, most likely due to the unique tonsillar crypt epithelium. Terms such as ‘branchial cleft cyst’ and ‘branchial cleft cyst carcinoma’ are confusing and misleading, and both should be abandoned.
